# c-Jun N terminal kinase modulates NOX-4 derived ROS production and myofibroblasts differentiation in human breast stromal cells

**DOI:** 10.1186/1471-2407-14-640

**Published:** 2014-08-30

**Authors:** Nicolás Tobar, Marcela Toyos, Carla Urra, Nicolás Méndez, Rodrigo Arancibia, Patricio C Smith, Jorge Martínez

**Affiliations:** Laboratorio de Biología Celular y Molecular, INTA, Universidad de Chile, El Líbano 5524, Macul, Santiago, Chile; Laboratorio de Fisiología Periodontal, Facultad de Medicina, Pontificia Universidad Católica de Chile, Santiago, Chile

**Keywords:** Fibrosis, NOX-4, JNK, ROS

## Abstract

**Background:**

Hard consistency, developed under the influence of tumor cell factors, is a characteristic feature of a breast tumor. Activation of resident fibroblasts leading to a myofibroblast phenotype is the principal feature that orchestrates this fibrotic process. The aim of this study was to assess the effects induced by TGF-β1, a growth factor abundantly present in tumor microenvironment, on the molecular mechanisms that mediate myofibroblastic differentiation of normal human mammary fibroblasts.

**Methods:**

We used an immortalized fibroblastic cell line derived from normal mammary tissue (RMF-EG cells) to study the effect of TGF-β1 in the expression of α-SMA and CTGF as markers of myofibroblastic differentiation. The influence of redox status and JNK activity on TGF-β1-induced transcriptional activity was measured by a luciferase reporter assay. We also used a shRNA approach to evaluate the influence of NOX4 in myofibroblastic differentiation.

**Results:**

TGF-β1 stimulates the expression of myofibroblast markers α-SMA and CTGF. Using a NOX inhibitor (DPI) and cells expressing a shRNA for NOX4, we demonstrated that TGF-β1 promotes an oxidative environment that favors myofibroblastic differentiation. We also found that activation of c-Jun N-terminal kinase is required for TGF-β1-dependent expression of CTGF, NOX4 and α-SMA.

**Conclusions:**

Human mammary stromal fibrosis, evaluated by the expression of early and late markers as CTGF and α-SMA, depends on the activation of JNK signaling pathway. Our results show that JNK activation is an early event that precedes the increase in ROS levels leading to myofibroblastic differentiation and tumor fibrosis, suggesting that inhibition of JNK may be used a method to interrupt the development of tumor desmoplasia.

## Background

Interactions between stromal fibroblasts and migratory cells at the primary site of tumors create a supportive microenviroment for cancer growth and survival, evasion of immune surveillance and metastatic potential [1, 2]. Given their abundance in the tumor site and the variety of functions described in the literature, tumor fibroblasts have been proposed as key players in the acquisition of malignant properties by carcinoma cells [3].

It is currently known that cells of the tumor parenchyma and stroma are engaged in an active crosstalk, and that the composition of the stroma and the nature of tumor-stromal interactions reciprocally change over time together with tumor progression [4]. Thus, stromal soluble factors can modify the invasive potential of carcinoma cells, in turn epithelial features are responsible for the stromal desmoplastic response that characterizes some types of tumors [5]. On the other hand, it has also been demonstrated that the normal microenvironment functions as a non-permissive barrier to block tumor initiation and/or progression [6]. The protective role of normal stroma was also confirmed in a model of human breast morphogenesis in which authors found that this process also depends on the involvement of a proper fibroblastic stroma [7].

Several studies provide evidence that oxidative stress, produced by an excessive production of Reactive Oxigen Species (ROS), constitutes an effective environmental stimulus for tumor progression [8]. ROS exerts a key role in a variety of processes associated with epithelial malignancy such as cell proliferation, epithelial-mesenchymal transition (EMT), angiogenesis, apoptosis evasion and enhancement of metastatic potential [9]. Endogenous generation of ROS arises from two main sources: mitochondria and the NAD(P)H oxidase (NOX) system [10]. In non-phagocytic cells, the NOX family is a key component of the so-called “redox signaling system” which regulates many cellular responses by modulating the intracellular ROS content. Previous work of our group showed that an enhancement of stromal NOX-4 expression and the subsequent increase of intracellular ROS production stimulated by TGF-β1 contained in an epithelial conditioned medium, constitutes a permissive element in the acquisition of migratory properties by carcinoma cells [11].

TGF-β1 has been identified as one of the main tumor-derived soluble factor that alters the stroma toward cancer progression by promoting the differentiation of stromal fibroblasts to a myofibroblastic phenotype [12]. The prevalence of myofibroblasts in the cancer microenvironment has been shown in many different types of cancer including colon, liver, lung, prostate, ovary, pancreas, and breast [13]. This “activated” form of stromal fibroblasts allows the growth in volume of fibrotic tumors, enhances contractile properties and provokes changes in the extracellular matrix (ECM) composition [14]. Contractibility is mainly achieved by the incorporation of α-smooth muscle actin (α-SMA) into stress fibers, molecules whose expression is controlled by the joint action of growth factors like TGF-β, specialized ECM proteins like the fibronectin splice variant ED-A FN and mechanical forces derived from changes in ECM composition [15].

Increasing evidence indicates that the rather linear view through which TGF-β signaling occurs by the single activation of Smad pathway, does not account for the variety of responses obtained under TGF-β stimulation. The emergence of the concept of non-Smad-TGF-β signaling has provided a more complete understanding of the wide and diverse spectrum of TGF-β cellular responses [16]. The molecular mechanisms underlying the profibrotic effects of TGF-β is a good example of this dilemma. In spite of several studies conducted in many different cellular systems, using different experimental approaches, the specific mechanism that accounts for TGF-β-dependent expression of fibrotic markers, has not been fully elucidated. Thus, an important area of study should consider the relative importance of the canonical and non-canonical pathways in TGF-β-dependent fibrogenic gene responses [17].

In the present work, using a stromal cell line derived from normal breast tissue, we show that TGF-β1-dependent expression of fibrotic markers, such as α-SMA and connective tissue growth factor (CTGF), depended on the establishment of a redox threshold reached by the early expression of NOX-4. Moreover, we also demonstrate that c-jun N-terminal kinase (JNK) activation constituted a key step for the expression of both NOX-4 and fibrotic markers putting the TGF-β-dependent activation of this route as an initial event during the breast stromal myofibroblasts differentiation process.

## Methods

### Cell culture, cell line and chemicals

Human cell line RMF-EG [7] was a generous gift from Dr. Charlotte Kupperwasser (Tufts University, MA). These cells were cultured in DMEM/F12 (Invitrogen Carlsbad, CA), supplemented with 10% fetal bovine serum (FBS) (Hyclone, Logan UT) and maintained in a humidified atmosphere of 37°C, 5% CO2. Human Recombinant TGF-β1 was purchased from R&D Systems (Minneapolis, MN). SP600125 and SIS3 were purchased from Merck (Darmstadt, HD, DE). Diphenyleniodonium chloride (DPI) and 2′,7′-dichlorodihydrofluorescein diacetate (H2DCFDA) were acquired from Sigma Aldrich (St. Louis MO).

### Treatments, western blot and antibodies

RMF-EG cells were seeded in 60 mm dishes and pretreated 1 hour before the onset of TGFβ-1 stimulus with: 2.5 μM DPI, 10 μM SP600125 or 3 μM SIS3, in order to inhibit ROS production, JNK or Smad3 activity, respectively.

After the corresponding treatments, cells were resuspended in lysis buffer (50 mM HEPES, pH 7.4, 150 mM NaCl, 2 mM MgCl_2_, 2 mM EGTA, 1% Triton X-100 and 10% glycerol) supplemented with complete protease inhibitors (Roche, Mannheim). Protein concentration was determined by the Bradford method (Fermentas, Maryland). Protein extracts were heat denatured in SDS-Sample buffer (240 mM Tris–HCl, pH 6.8, 8% SDS, 40% glycerol and 20% 2-mercaptoethanol). Equal amounts of protein from different treatments were resolved by SDS-PAGE in 10% polyacrylamide gels and electrotransferred to polyvinylidenedifluoride (PVDF) membranes using a buffer containing 24 mM Tris, 194 mM glycine and 20% methanol. Proteins were further analyzed using the ECL chemiluminescence detection kit (Amersham, Arlington Heights, FL). The immunoreactions were achieved by incubation of the membranes, previously blocked with a solution containing 5% BSA in Tris-buffered saline (TBS) and 0.05% Tween 20 (Sigma, St. Louis, MO), with the following antibodies: rabbit anti-phospho (Thr183/Tyr185) JNK 1,2 (#4668) was from Cell Signaling Technology (Danvers , MA), rabbit anti-JNK1,2 (sc-7345) and goat anti-CTGF (sc-14940) were from Santa Cruz (Santa Cruz, CA), rabbit anti-NOX-4 (ab81967), mouse anti-α-SMA (ab7817) and rabbit anti-Smad3 (phospho S423 + S425) antibodies (ab52903) were from Abcam (Cambridge, MA) and mouse anti-Smad3 (MA5-15663) from Pierce Biotechnology (Rockford IL.). Secondary antibodies against mouse, rabbit and goat conjugated to peroxidase were purchased from Rockland Immunochemicals (Gilbertsville, PA). Densitometric analysis of western blot bands was performed using Molecular Imaging Software, version 4.0 of Kodak (Rochester, NY).

### Reporter assay

Reporter assays using TGF-β-responsive promoter 3TP-Luc constructs (kindly donated by Dr. E. Brandan, Pontificia Universidad Católica de Chile) were performed as described previously [18], in cells under the stimulus of TGF-β1 in the presence or absence of DPI or SP600125, as indicated above. All transient transfection experiments were carried out using TransIT-2020 reagent as vehicle for plasmids according to manufacturer instructions (Mirus, Madison, USA). Relative luciferase units were determined using a Dual Luciferase Reporter Assay System kit (#E1960 from Promega, Madison WI) in a Berthold FB12 luminometer. The pRL-tk Renilla luciferase vector (Promega, Madison WI) served as internal control to correct for transfection efficiency and normalization.

### Measurement of intracellular redox state

The oxidation-sensitive fluorescent probe 2′,7′-dichlorodihydrofluorescein diacetate (H2DCFDA) was used to analyse the total intracellular content of ROS [11]. In a representative assay, RMF-EG cells were incubated with 5 μM H2DCFDA in serum- and phenol red-free medium (Gibco Invitrogene, CA) for 30 min at 37°C. Cells were then washed and lysed with 0.1 N NaOH, and fluorescence was monitored using a microplate fluorometer (Spectra MAX, Gemini EM, Molecular Devices) with wavelengths of 480 and 530 nm for excitation and emission, respectively [19]. In experiments in which ROS production was exogenously modulated, RMF-EG cells were pre-treated for different periods of time with 5 ng/ml of TGF-β1 in presence or absence of 10 μM SP600125 before fluorometric determination of ROS.

### Quantitative PCR

Total RNA was isolated from control and TGF-β1-stimulated RMF-EG cells, treated or not with 2.5 μM DPI or 10 μM SP600125, with Trizol (GIBCO) according to the manufacturer’s instructions. mRNA expression was assessed by real time PCR using a Light Cycler instrument (Roche, Germany). The reaction was performed using 100 ng of cDNA and LightCycler®FastStart DNA Master SYBR Green I kit (Roche) in a final volume of 20 μL. All the reactions were performed in duplicate, and negative controls were included. The primers used were: NOX4, forward: 5′TAGATACCCACCCTCCCG3′, reverse: 5′TGGGCTCTTCCATACAAATC3′, PCR product size: 169pb; α-SMA, forward: 5′ GCCGACCGAATGCAGAAGGA 3′, reverse: 5′ TGCGGTGGACAATGGAAGGC3′, PCR product size: 190pb; CTGF, forward: 5′TTGGCCCAGACCCAACTATG3′, reverse: 5′ CAGGAGGCGTTGTCATTGGT3′, PCR product size: 240pb and GPDH, forward 5′ CAAAATCAAGTGGGGCGATGCTG 3′, reverse 5′ TGTGGTCATGAGTCCTTCCACGAT 3′. In every case, mRNA expression was normalized by viable cell number at the end of the experiment and using GAPDH as loading control.

### Lentivirus production and infection of breast stromal cells

HEK293FT cells were grown in 60 mm culture plates to 80–90% confluence; Lipofectamine 2000 reagent (Invitrogen) was used to transfect cells (following the manufacturer’s instructions) with the pVSVg, pΔ8.9, and pLKO.1-shRNA plasmids (TRCN46088 and TRCN46090 to NOX4 and TRCN1056 and TRCN1013 to JNK1 and 2 respectively, Sigma-Aldrich, St Louis MO) at a 1:2:3 ratio, respectively, with a maximum total DNA of 10 μg. pLKO.1 non-target shRNA (Sigma-Aldrich, St Louis MO) was used as a control. After 16–18 h, the culture medium was replaced and cells were maintained at 37°C for 48 h. Supernatants containing pseudo-typed particles were collected, filtered through a PVDF filter (0.45 μm pore size), and concentrated by centrifugation at 3800 × g for 30 min at 4°C in an Amicon Ultra-15 centrifugal filter device (100 K, Merck Millipore) according to the manufacturer’s instructions. Aliquots of concentrated viral particles were immediately stored at − 80°C. RMF-EG cells were plated in 60 mm culture plates and infected for 48 h with 40 μl lentiviral particles containing shRNA-NOX4 or JNK1,2 or control plasmids. Afterwards, cells were stimulated with 5 ng/ml TGF-β1 for 24 h and lised for western blot analysis.

### Immunofluorescence analysis

RMF-EG cells (5x10^4^) were plated on glass coverlips (24 h) and then incubated for 1 h with 2,5 μM DPI or 10 μM SP600125 previous to a 48 h incubation with 5 ng/ml of TGF-β1 in serum-free medium. After PBS washing, cells were fixed with 4% paraformaldehyde in PBS, permeabilized with 0.1% Triton X-100 in PBS and processed for immunofluorescence as previously described [20]. Primary mouse monoclonal antibody directed to human α-SMA (ab7817) was from Abcam (Cambridge, MA) and the fluorescence secondary antibody was form Invitrogen, Molecular Probes, Carlsbad, CA. Nuclei were stained with Hoescht (Invitrogen Molecular Probes). Coverlips were mounted on glass slides and images were collected by immunofluorescence microscopy with a digital camera (Carl Zeiss, Dresden, Germany).

### Knocking down of Smad2/3 pathway by siRNA

To investigate whether TGF-β1-dependent activation of Smad2/3 pathway plays a role in NOX4 expression, we evaluated mRNA expression of NOX4 after knocking down Smad2/3. We used a commercial pool of small interfering RNAs directed to this molecule (Santa Cruz, sc-37238 and sc-37007 as a control) and used the TransIT-siQUEST transfection reagent (Mirus) according to manufacturer instructions.

### Statistical analysis

Experimental data are presented as mean ± standard error and were performed in triplicate. Data were subjected to a variance analysis; comparisons among groups was performed by Anova-Tukey using the GraphPad Prism 5 software (GraphPad Software, San Diego, CA). A value of p < 0.05 was considered statistically significant.

## Results

### TGF-β1-stimulated NOX4 and CTGF expression and intracellular ROS production preceded α-SMA expression

**Figure 1:**
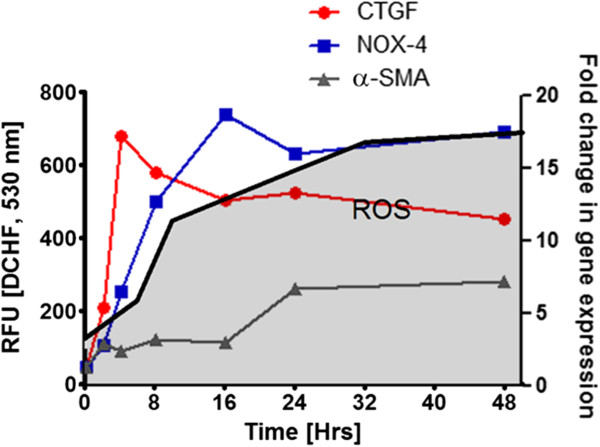
**TGF-β1 increased intracellular ROS production and CTGF, NOX-4 and α-SMA mRNA expression.** Time-course evaluation of CTGF, NOX-4 and α-SMA mRNA expression performed by qPCR. RMF-EG cells cultures, previously deprived of serum were stimulated with 5 ng/ml of TGF-β1 and their mRNA levels were evaluated at the indicated times. In a parallel group of cells, intracellular ROS production was assessed by measuring fluorescence generated after H_2_DCFDA incubation. Figure is representative of three independent experiments. Values were normalized by number of viable cells at the end of the experiment.

To analyze whether TGF-β1 exerts its stimulus on the expression of molecules relevant to myofibroblastic differentiation in a sequential manner, we performed a quantitative PCR (qPCR) analysis of α-SMA, CTGF and NOX4 mRNA expression in a time range from 2 to 48 h. During the same time range we analyzed the intracellular ROS production using H2DCFDA as a probe. Under a 5 ng/ml TGF-β1 stimulus, CTGF reached an early expression maximum around 4 h that was maintained throughout the time period (Figure 1). NOX4 expression increased in a linear manner reaching its expression peak around 16 h. On the other hand, α-SMA displayed a later expression, reaching its peak around 24 h. Intracellular ROS production increased in expression from 4 to 16 hours, at which time its maximum level was reached (Figure 1).

### Expression of myofibroblast markers stimulated by TGF-β1 was attenuated by NOX inhibiton

**Figure 2:**
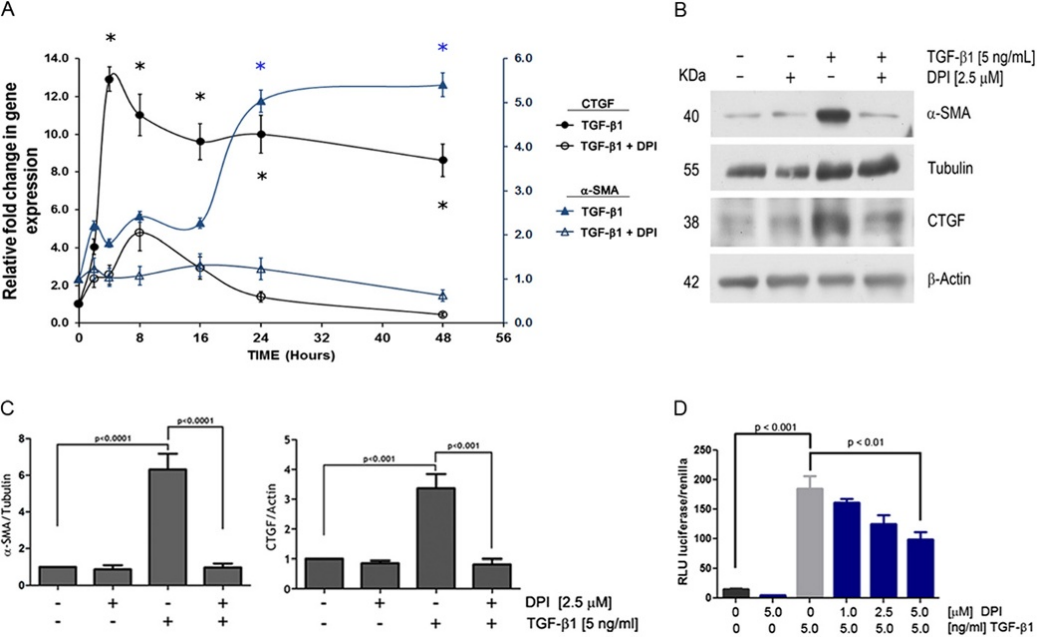
**NADPH oxidase activity modulated the expression of CTGF and α-SMA. A.** Inhibition of NOX activity decrease CTGF and α-SMA expression. Time-course expression of CTGF and α-SMA mRNA evaluated by qPCR in 5 ng/ml TGF-β1- stimulated RMF-EG cells pre-treated or not with DPI. **B.** DPI inhibited TGF-β1-induced CTGF and α-SMA protein expression. RMF-EG cells were treated with TGF-β1 (5 ng/ml) in the presence or absence of 2,5 μM DPI. Protein expression of CTGF and α-SMA was evaluated by western blot after 24 h and 72 h, respectively. **C.** Densitometric analysis of western blots bands in B. Columns represent the mean of α-SMA to Tubulin signal and CTGF to Actin signal from three independent experiments; bars ± SE **D**. DPI inhibits TGF-β1-induced 3TP-luc luciferase reporter activity. RMF-EG cells transiently transfected with 3TP-luc luciferase construct and were pre-treated or not with DPI before stimulation with TGF-β1 for 16 h. Values are average of three separate experiments performed in triplicate.

In non-phagocytic cells, the NOX family of enzymes constitutes a key element providing the so-called “redox signaling system” which regulates many cellular functions [21]. To test whether intracellular redox level controlled by NOX was able to modify the TGF-β1-dependent expression of canonical myofibroblast markers in breast stromal cells, we subjected RMF-EG cells to a 1 hour pretreatment with 2.5 μM of the flavoprotein inhibitor diphenyleniodonium chloride (DPI), previous to TGF-β1 stimuli. The time-course mRNA expression of α-SMA and CTGF in the presence or absence of DPI displayed inhibition at all time points (Figure 2A). When the same phenomenon was analyzed in terms of protein expression, (Figure 2B) a very similar picture arose, revealing a significant inhibition of the expression of both markers in the presence of DPI. After correction for cell viability, differences between control and DPI-treated cells remained significant (Figure 2C). To analyze whether this inhibition was the result of a blockade of the TGF-β1 transcriptional activity, we performed a 3TP-luc reporter assay to measure the effect of DPI in ALK5-Smad2/3 pathway activation. Preincubation of RMF-EG cells with DPI generates a dose-dependent inhibition of reporter activity (Figure 2D).

### NOX4 knockdown blocked TGF-β1 stimulus on α-SMA expression

**Figure 3:**
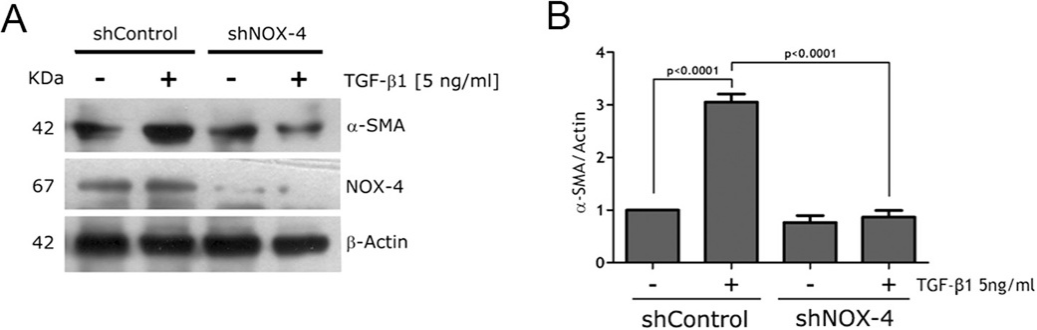
**NOX-4 knockdown reduced α-SMA expression in response to TGF-β stimulus. A.** RMF-EG cells were infected with viral particles (prepared as described in Material and Methods) carrying a sequence specific shRNA targeting NOX4 or a non-related sequence. After an infection period of 48 h, in the presence or absence of 5 ng/ml of TGF-β1 proteins were analyzed by western blot to a-SMA and NOX4. **B.** Columns represents mean ± SE of α-SMA signal to Actin from densitometric analysis of three independent experiments.

Using RMF-EG cells we have previously demonstrated that production of ROS in response to TGF-β1 was mainly accomplished by a previous increase on NOX-4 expression [11]. To test whether the ROS-dependent stimulus on α-SMA protein expression was attributable to a TGF-β1 stimulus on NOX4 expression/activity, we used a lentiviral based shRNA strategy to knock down NOX4. In doing so, we infected for 48 h RMF-EG cells with viral particles (prepared as described in Material and Methods) carrying a sequence specific shRNA targeting NOX4. As Figure 3 shows, RMF-EG lentivirus infected cells expressed a diminished level of NOX-4, which in turn, was able to impair TGF-β1 induced α-SMA protein expression.

### TGF-β1 stimulated JNK activation, independent of Smad3

**Figure 4:**
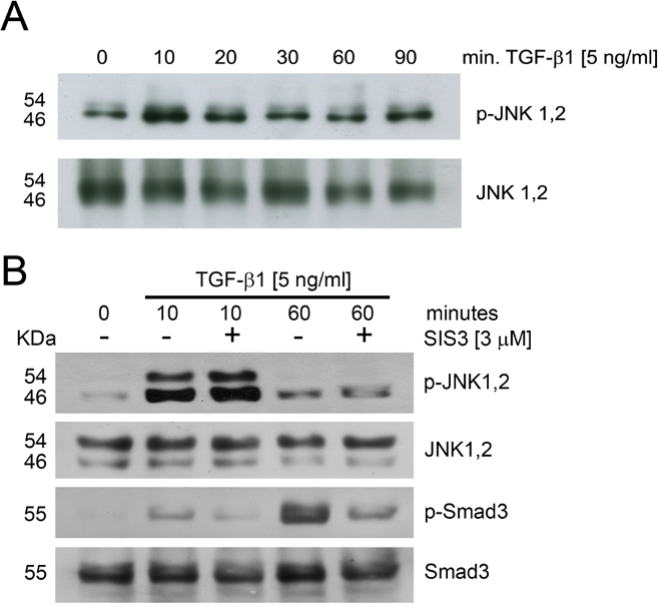
**TGF-β-dependent activation of c-JNK was independent of Smad3 activation. A.** Serum-deprived RMF-EG cells were incubated with 5 ng/ml of TGF-β from 10 to 90 min. The levels of phosphorylated and total JNK were evaluated by western blot. **B.** RMF-EG cells were pre-treated with 3 μM SIS3, specific inhibitor of Smad3, and stimulated with 5 ng/ml TGF-β as in B. c-JNK and Smad3 activation were evaluated by western blot.

It has been demonstrated that intracellular TGF-β1 signaling occurs by the activation of an array of signaling routes. Among them, Smads and c-Jun N-terminal kinase (JNK) seem to be the most prominent and probably exhibit some level of interaction between each other [22]. To evaluate the activation of the JNK pathway by TGF-β1 we examined the phosphorylation pattern of JNK in a time-course assay. Figure 4A shows that TGF-β1 induced an early activation of the route with a peak around 10 min and a return to the basal state through the remaining time of stimulus. To investigate whether a previous activation of Smad3 was required for JNK activation, cells were pre-treated with SIS3, a specific inhibitor of Smad3, prior to TGF-β stimulus. As Figure 4B shows, JNK phosphorylation was not affected, neither at 10 nor 60 minutes, suggesting that JNK activation was, in fact, independent of previous Smad3 activation regardless of time.

### JNK inhibition prevented the expression of myofibroblastic markers

**Figure 5:**
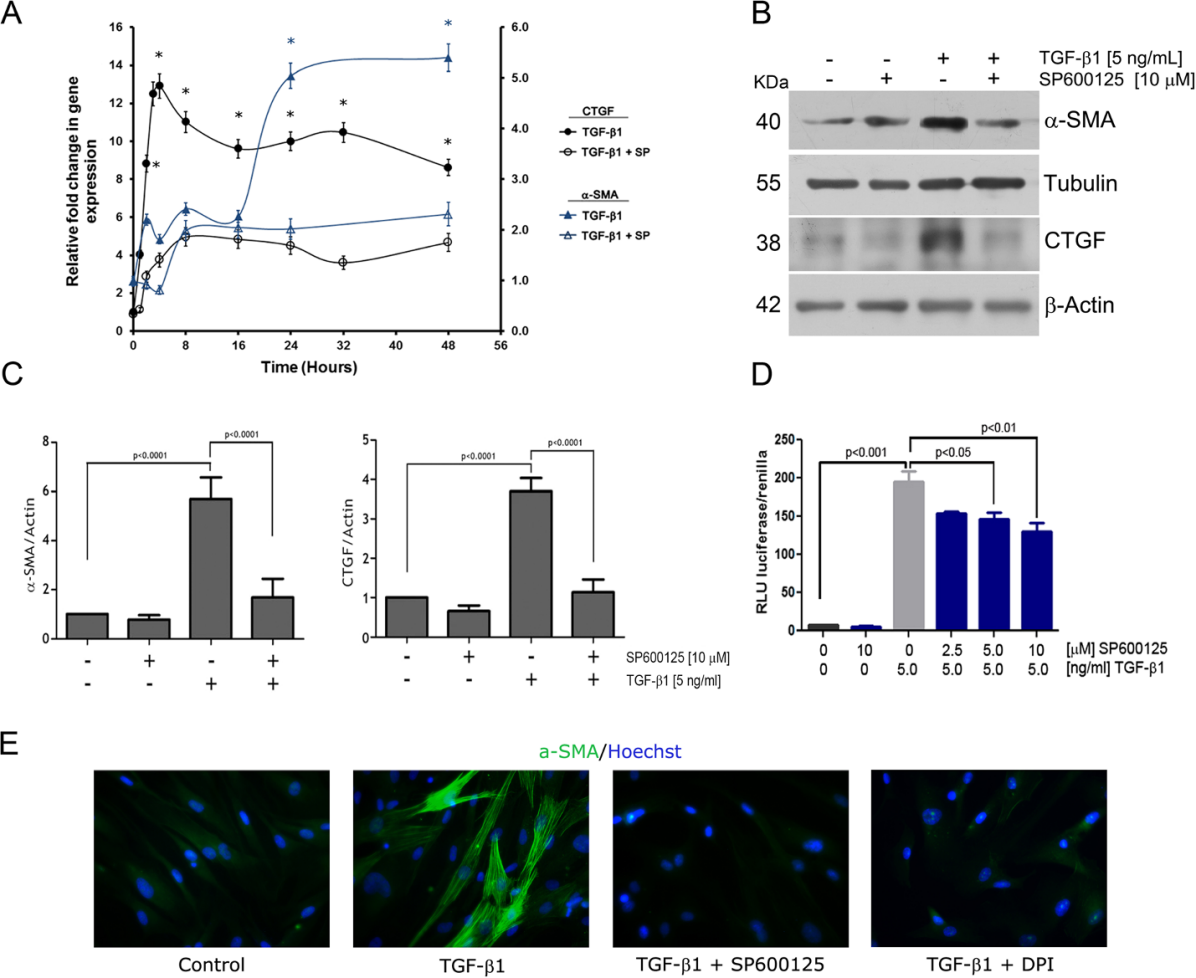
**Inhibition of c-JNK activity reduced α-SMA/CTGF expression and partially reduced TGF-β-dependent transcriptional activity. A.** CTGF and α-SMA mRNA time-course expression in RMF-EG cells. Serum-deprived RMF-EG cells were pre-treated or not with 10 μM SP600125 for 16 h and further stimulated with 5 ng/ml of TGF-β1 at the indicated times. mRNA expression of both genes were evaluated by qPCR. **B.** RMF-EG cells were treated with TGF-β1 (5 ng/ml) in the presence or absence of 10 μM SP600125. Protein expression of CTGF and α-SMA was evaluated by western blot after 24 h and 72 h respectively. **C.** Densitometric analysis of western blot bands in **B**. Columns represents mean ± SE ratio of α-SMA to Tubulin and CTGF to Actin signal from three independent experiments. **D.** RMF-EG cells were transiently transfected with 3TP-luc luciferase construct and then pre-treated or not with SP600125 before stimulation with TGF-β1. Values presented are representative of three separate experiments performed in triplicate. **E.** Immunofluorescence analysis to α-SMA of control and TGF-β1-treated (48 h) RMF-EG cells preincubated in the presence or absence of DPI (2,5 μM) or SP600125 (10 μM). Figure is a representative image of an experiment performed in duplicate.

To evaluate whether the TGF-β1-stimulated JNK activation had a functional consequence in the expression of myofibroblast markers, we preincubated RMF-EG cells with SP600125, specific inhibitor of JNK, prior to TGF-β1 stimulus. As Figure 5A, B and C show, both α-SMA and CTGF mRNA and protein expression were inhibited in the presence of the JNK inhibitor. To test whether the SP600125 inhibition of α-SMA and CTGF expression operates via the reduction of TGF-β1 transcriptional activity, we used the 3TP-luc reporter assay to measure the effect of SP600125 in the ALK5-Smad2/3 pathway activation. As Figure 5D shows, incubation of RMF-EG cells with SP600125 prior to TGF-β1 stimulus, generated a reporter activity inhibition. Considering that c-Jun expression is also under the control of JNK [23], our data may suggest that SP600125 exerts its action by blocking the activation of the AP-1 site of 3TP-luc reporter. To confirm that SP600125 was able to inhibit α-SMA expression, we performed an immunofluorescence analysis in RMF-EG cells treated with TGF-β1. Figure 5E shows that in the presence of the inhibitor there was a negligible expression of α-SMA in cells treated with TGF-β1. A similar result was obtained when cells were pre-treated with 2.5 μM DPI reinforcing the suggestion that a NOX activity is involved in α-SMA expression as shown in Figure 2.

### JNK activity controlled intracellular redox status through modulation of NOX4 expression

**Figure 6:**
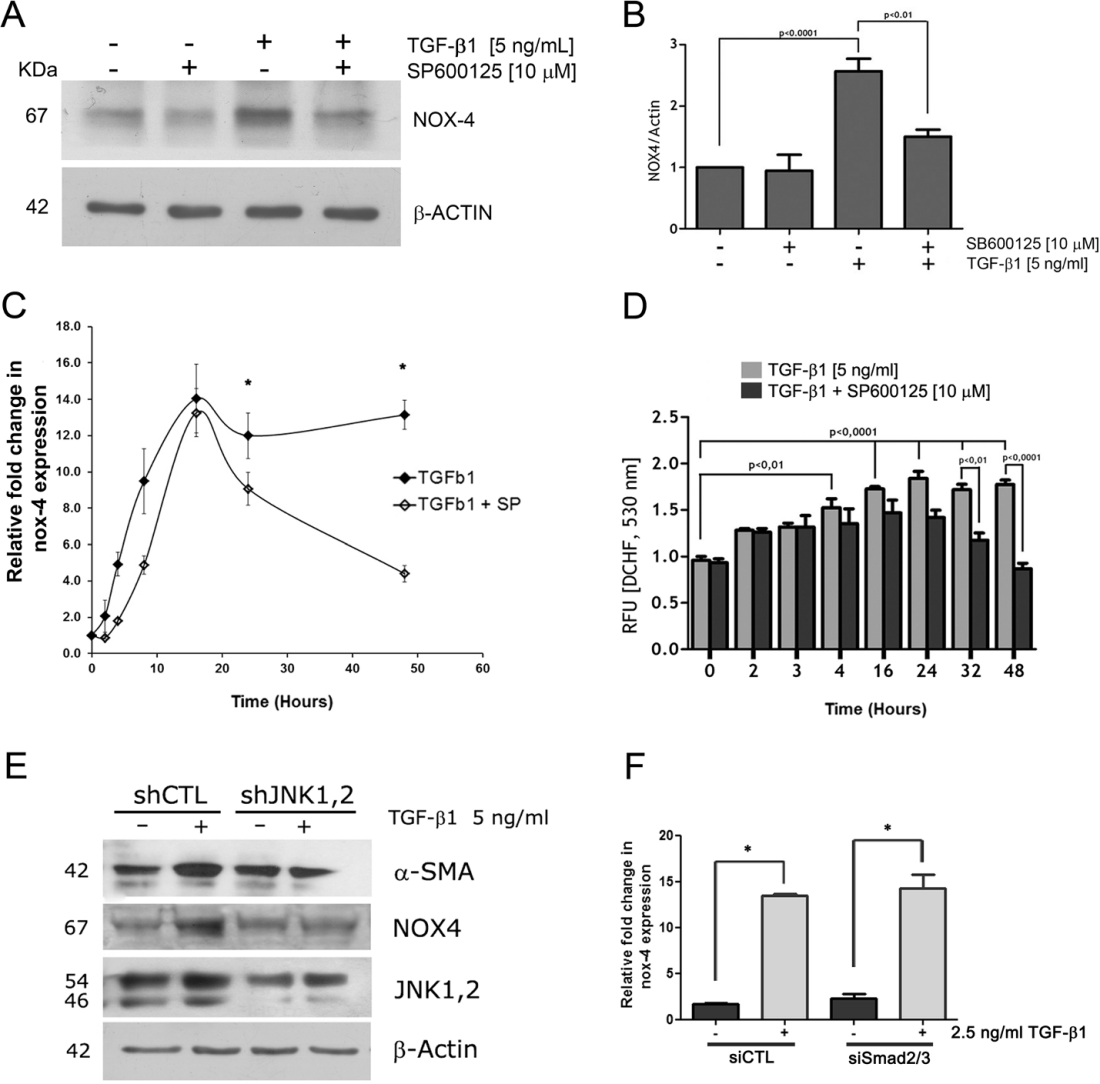
**NOX-4 expression and intracellular ROS production depended on c-JNK activity. A.** Serum-starved RMF-EG cells were pretreated or not with 10 μM SP600125 for 16 h and stimulated for 24 h with 5 ng/ml of TGF-β1. NOX-4 protein expression was evaluated by western blot. **B.** Densitometric analysis of western blot bands in A. Columns represents mean ± SE ratio of NOX-4 to Actin signal of three independent experiments. **C.** Time-course of NOX-4 mRNA expression in TGF-β1-stimulated RMF-EG treated or not with SP600125 measured by qPCR. **D.** Time-course of intracellular ROS production evaluated by fluorescence generated with H_2_DCFDA incubation in TGF-β1-stimulated RMF-EG cells pre-treated or not with 10 μM SP600125. Values are representative of three separate experiments. **E.** RMF-EG cells were infected either with viral particles carrying a sequence specific shRNA targeting JNK1,2 (shJNK1,2) or a non-related sequence (shCTL) and treated or not with 5 ng/ml of TGF-β. α-SMA, NOX4 and JNK protein expression was evaluated by western blot. Figure is a representative result of an experiment performed in duplicate. **F.** Smad2/3 knocking down does not affect NOX4 expression. RMF-EG cells (3x10^5^) transfected with a siRNA directed to Smad2/3 were stimulated with 2.5 ng/ml of TGF-β1 for 24 h and mRNA expression was analyzed by qPCR.

To test whether the JNK activation played a significant role on the acquisition of the intracellular redox level needed for myofibroblast markers expression, we used two different experimental approaches. First, we analyzed the effect of JNK inhibitor on both, NOX4 protein expression and intracellular ROS production. Figure 6A and B show that pre-incubation of RMF-EG cells with 10 μM SP600125 produced a reduced NOX-4 protein expression that correlated with mRNA expression in a time-course experiment (Figure 6C). In the same time-course approach, pre- treatment of RMF-EG with SP600125 provoked a decreased production of H2DCFDA-reactive intracellular ROS, (Figure 6C) which becomes evident after 24 h of TGF-β1 stimulus, period in which NOX-4 expression reached its maximum level as is shown in Figure 1. Secondly, we knocked down the expression of JNK using a lentiviral strategy using a shRNA directed to JNK1 and 2. Figure 6D shows that RMF-EG cells expressing the shRNA did not respond to TGF-β stimulus, displaying a decreased expression of α-SMA and NOX4. To test whether Smad2/3, the canonical TGF-β signaling transducer, participated in NOX4 expression, we performed a knocking down of Smad2/3 using a siRNA approach. As Figure 6F shows, under this experimental condition, the expression of NOX4 was not affected, reinforcing our interpretation of the role of JNK in NOX4 expression.

## Discussion

Breast tumours belong to a group of neoplastic lesions which, under the influence of carcinoma cell products, originate a fibrous structure responsible for the hard consistency of the tumoral mass [24]. This fibrotic response is largely due to the activity of TGF-β, a growth factor that directly induces the transdifferentiation of fibroblasts into myofibroblasts [12]. Results from this work support the hypothesis that TGF-β1 plays a critical role in the development of breast fibrosis. Our findings indicate that this activity depends first on the activation of c-Jun terminal kinase and the subsequent NOX4-dependent ROS production.

Using an immortalized cell line derived from normal breast tissue, we demonstrated that TGF-β1 induces a sequential expression of CTGF, NOX-4, and α-SMA. These consecutive events also involve an increase in TGF-β1-dependent ROS production. The inhibition of ROS production by DPI suggests that at least a fraction of this redox level depends on a NAD(P)H oxidase activity, the target of the inhibitor. The blocking of NOX activity produced an inhibition of the expression of myofibrotic markers (shown in Figure 2), where CTGF and α-SMA expression were strongly inhibited by DPI at mRNA and protein levels after 24 h of TGF-β1 stimulus. Previous work, also performed in fibroblastic cells, has suggested that NOX-4 is the isoform responsible for the generation of the redox environment that, in turn, favours myofibroblastic differentiation [25, 26]. Prior work of our research group found that NOX-4 is the only isoform of NOX expressed in RMF-EG cells that is subjected to epithelial control [11]. In this stromal cellular system, NOX-4 was in fact involved in the expression of fibrotic markers. Considering that TGF-β-dependent NOX-4 expression is an early event in the TGF-β-dependent myofibroblastic differentiation, it is possible that the establishment of a redox threshold is a prerequisite for the long-term myofibroblastic process. It has been previously demonstrated that TGF-β1 was able to induce mitochondrial ROS production at early times and stimulated CTGF gene transcription at 24 h [27]. Our results showing that DPI was able to block CTGF mRNA expression as early as 4 h post stimulus can be explained because DPI is a fairly unspecific drug that inhibits both NOX4 and mitochondrial ROS production [28]. Therefore, it is possible that, at early time points, CTGF gene expression was stimulated by a slight increase in TGF-β1-induced mitochondrial ROS and later, ROS production and CTGF expression was sustained by NOX4 activity.

Searching for a molecular target of the TGF-β→NOX-4→ROS signaling axis, the nuclear MAPK phosphatase-1 (MKP-1) has been proposed as a direct oxidizable molecule and that its reduced phosphatase activity results in an induction of JNK/p38 phosphorylation and PAI-1 expression [29]. In agreement with these results we found that activation of 3TP-luc, reporter construct that displays a ~400-nucleotide region of the PAI-1 promoter, is also sensitive to ROS, given that its activity decays in the presence of DPI in a dose-dependent manner (Figure 2).

In spite of the fact that many of the transcriptional responses to TGF-β are mediated by the activation of Smad proteins, it has been demonstrated that these responses differ depending on the cell type and cross-talk between Smad signaling and other signaling systems [30]. Thus, during the last decade, growing evidence has supported the idea that non-Smads pathways actively participate in TGF-β signaling [31]. Some of the main alternative pathways activated by TGF-β in the context of cancer progression are the p38 and c-Jun N-terminal kinase (JNK), pathways belonging to the MAPK family [25]. Using a specific inhibitor of JNK, our results show that the TGF-β-dependent stimulus on fibrotic markers in RMF-EG cells (at mRNA and protein levels) depended on the factor-dependent activation of JNK. Our results suggest that inhibition of JNK by SP600125 blocked TGF-β-dependent early CTGF expression in a ROS-independent manner. However, after 16 h, JNK inhibition diminished ROS production simultaneously to inhibit NOX4 expression (Figure 6D and C) suggesting that, at this time, NOX4 activity sustains ROS production in a JNK-dependent manner.

It has long been recognized that intracellular production of ROS activates JNK and, in many cases, a specific requirement of NOX activity has been demonstrated [32]. For example, using prostatic fibroblasts, Sampson et al. have proposed that TGF-β-dependent JNK activation requires a prior stimulus on NOX-4, putting the NOX-4 expression (and the subsequent ROS production) as the initial step in a cascade of events that ends in myofibroblastic differentiation. Our results, from breast stromal cells, offer an alternative understanding. Results in Figure 6 show that NOX4 protein expression and ROS production are clearly affected by both pharmacological inhibition and knocking down of JNK, putting this signaling pathway, and not the canonical Smad2/3 route (Figure 6F), as the initial signal of a sequential mechanism that ends in tumor fibrosis. In addition, other studies have proposed that JNK is involved in fibrotic response. In fact, it has been described that, under the effect of TGF-β, CTGF expression and corneal wound healing in vivo are mediated by JNK activation [33]. Moreover, CC-930, a selective inhibitor of JNK, which is in a clinical trial as an antifibrotic drug (http://www.clinicaltrials.gov; trial identifier NCT01203943), has been shown to exert a potent antifibrotic effect, preventing myofibrolasts differentiation and the accumulation of collagen in both in vitro and in vivo models of systemic sclerosis [34].In conclusion, our results suggest that JNK activation is an early and critical step in the TGF-β1-dependent myofibroblastic differentiation process in normal human breast fibroblasts. Stimulation of JNK causes a sequential transcriptional activation that promotes an early ROS-independent stimulation of fibrotic markers such as CTGF (Figure 5A and Figure 6D) and, at later time points, the establishment of a NOX-4-dependent long-term α-SMA expression, which generates a pro-fibrotic microenvironment that promotes cancer progression.

## Conclusion

These results provide experimental evidence supporting the hypothesis that TGF-β1-induced JNK activation precedes the factor-dependent increase in intracellular ROS production, leading to myofibroblastic differentiation and tumor fibrosis. We found that the activity and expression of JNK are uniquely involved in the expression of NOX4 and in early (e.g. CTGF) and late (e.g. α-SMA) fibrotic markers.

## Abbreviations

NOX-4: NADPH oxidase 4
ROS: Reactive oxygen species
TGF-β1: Transforming growth factor β1
α-SMA: α-smooth muscle actin
CTGF: Connective tissue growth factor
JNK: c-Jun N-terminal kinase
shRNA: Small hairpin RNA
DPI: Diphenylene iodonium
ECM: Extracellular matrix
ED-A FN: Extra domain-A fibronectin
EMT: Epithelial-mesenchymal transition
H2DCFDA: 2′,7′-dichlorodihydrofluorescein diacetate.
